# Analysis of the influencing factors of humanistic care ability of undergraduate nursing students in China

**DOI:** 10.1097/MD.0000000000048318

**Published:** 2026-05-01

**Authors:** Jia Li, Hai-xia Xu, Min Li, Yong-Li Wang, Ling-ling Li, Te-er Ba

**Affiliations:** a School of Nursing, Inner Mongolia Medical University, Hohhot, China; b Department of Radiotherapy, The Affiliated Hospital of Inner Mongolia Medical University, Hohhot, China; c School of Public Health, Inner Mongolia Medical University, Hohhot, China.

**Keywords:** analysis of influencing factors, cultivation strategy, humanistic care ability, undergraduate nursing students

## Abstract

Improving the ability of humanistic care can enhance the professional identity of nursing students and establish a harmonious nurse–patient relationship. This study aims to explore the current status and influencing factors of the humanistic care ability of undergraduate nursing students. The results of this study will provide the scientific basis for cultivating nursing students with high humanistic care ability, increasing clinical nurses, and improving nursing quality. This is a cross-sectional study. From September 2024 to November 2024, we conducted a questionnaire survey on 367 nursing undergraduate students from 3 medical colleges in China. Participants self-reported general demographic questionnaires, care ability assessment scales, emotional intelligence scales, and self-efficacy scales. The collected data were analyzed using various statistical methods, including descriptive analysis, independent sample *t*-tests, 1-way analysis of variance tests, and multiple linear regression analysis. All analyses were performed using the SPSS (IBM Corp., Armonk) software (version 27.0). *P* < .05 indicated that the difference was statistically significant. In this study, the humanistic care ability score of 367 undergraduate nursing students was (194.99 ± 33.31). Cognition is the dimension with the highest score, while patience is the dimension with the lowest score. Multiple regression analysis showed that emotional intelligence, gender, and self-efficacy were the main factors affecting undergraduate nursing students’ ability to provide humanistic care (*P* < .05). The humanistic care ability level of undergraduate nursing students in this study is at a moderate level. To improve this level, nursing educators should develop targeted intervention measures for various influencing factors. It is recommended that nursing education personnel strategically incorporate training on humanistic care abilities into practical teaching projects, stimulate nursing students to practice the virtue of humanistic care in their daily lives, and ultimately improve the quality of nursing services in clinical practice.

## 1. Introduction

With the aging population, the demand for healthcare continues to increase, but healthcare resources are tight, and nurses may primarily focus on treatment tasks in stressful work environments, sacrificing patient interaction. Therefore, patients often face medical staff who lack empathy and hold negative attitudes,^[1]^ leading to an increasing number of malignant injury incidents in China due to medical disputes. This serious lack of humanistic care reflects the lack of humanistic literacy and communication skills among some medical staff and also exposes the current situation of medical education in China that emphasizes technology over humanities. Medicine lacks humanity and is related to ongoing doctor–patient tensions and disputes, which have led to violent outcomes such as deaths and injuries among Chinese doctors.^[2,3]^ According to the “Guidelines for the Construction of Ideological and Political Education in Higher Education Curriculum,”^[4]^ medical education, especially nursing, emphasizes the dual requirements of integrating professional knowledge and skills with humanistic care abilities. Nursing students need to focus on their major and cultivate a humanistic spirit to achieve comprehensive development. This orientation aims to cultivate medical talents with both a profound academic foundation and noble professional ethics, ensuring that they can not only provide efficient professional services in practice but also treat every patient with empathy and humanistic care, thereby comprehensively improving the quality of medical services and patient experience.

Humanistic care ability is one of the core competencies of nurses, which is a prerequisite for establishing a harmonious nurse–patient relationship and can directly reflect the quality of nursing care. It refers to the ability to provide care services for patients, reflected in respect, understanding, and care for patients.^[5]^ Emotional intelligence reflects an individual’s general ability to perceive emotions, regulate emotions, and effectively respond to emotional situations.^[6]^ According to data from the National Health Commission,^[7]^ the total number of registered nurses in China will reach 5.63 million in 2023, with the proportion of those with a bachelor’s degree or above breaking through 50% for the first time. According to the plan jointly formulated by the Ministry of Education and the National Health Commission, it is expected that the number of registered undergraduate nurses will exceed 3.4 million by the end of 2024. The proportion of nurses is expected to reach 60% of the total number. Undergraduate nursing students are the reserve force for nursing work in medical institutions, and improving their humanistic care abilities is beneficial for them to provide high-quality nursing services to patients after entering clinical practice.

In 2020, a study conducted by foreign scholars showed that a holistic and patient-centered approach that focuses on individuals rather than diseases is advocated when implementing patient care.^[8]^ In recent years, research on the ability of humanistic care has become increasingly in-depth in China. Some scholars have pointed out that nursing students have a high level of humanistic care ability, which helps to meet the higher level needs of patients and improve their satisfaction.^[9,10]^ This is of great significance for improving the quality of nursing services and enhancing their professional identity.^[11]^ The professional identity of nursing students directly affects the number of clinical nurses and further influences the development of clinical nursing. Higher humanistic care ability has a positive impact on the professional identity of nursing students. In short, a higher level of humanistic care ability can enhance patients’ trust, support the long-term development of the nursing profession, and improve the quality of nursing services. Therefore, it is necessary for nursing educators to evaluate their ability to provide humanistic care for their students.

Previous research on humanistic care ability mainly focused on clinical nurses and was a multivariate-related study. However, there is a scarcity of in-depth investigation into the factors that affect the humanistic care ability of nursing students. Therefore, this study investigated and analyzed the current situation and influencing factors of 367 undergraduate nursing students’ humanistic care abilities, explored their training strategies, and the results of this study will lay the foundation for developing intervention models to improve nursing quality.

## 2. Materials and methods

### 2.1. Study design

This study used a cross-sectional design. The study complied with the strengthening of the reporting of observational studies in epidemiology checklist guidelines.

### 2.2. Setting and sample

This study utilized convenience sampling. From September 2024 to November 2024, we recruited nursing students from 3 provinces in China (Shandong Province, Shanxi Province, and Inner Mongolia Autonomous Region). The inclusion criteria include nursing students who voluntarily participate in the study and sign informed consent forms. The exclusion criteria include nursing students who are unable to participate due to various reasons, such as long-term vacation nursing students and absent nursing students. The Kendall sample size estimation method is used to determine the sample size based on a range of 5 to 10 times the number of survey variables.^[12]^ The survey includes 19 variables. Considering the low efficiency of 20% and potential loss of follow-up, the sample size for this study is determined to be between 119 and 238. In this study, all methods were approved by the Ethics Committee of Inner Mongolia Medical University Affiliated Hospital in accordance with the Helsinki Declaration of the World Medical Association (approval number: KY2025141). Additionally, our research is anonymous. During the investigation process, we only collected questionnaire information, not personal information.

### 2.3. Measures

#### 2.3.1. Demographic characteristics questionnaire

The demographic characteristics questionnaire was developed through a comprehensive literature review, group discussions, and expert consultation. This questionnaire consists of 11 items, including age, grade level, gender, home address, political affiliation, being an only child, serving as a class leader, participating in social activities, having hospitalization experience, reasons for choosing the nursing profession, and receiving humanistic care training.

#### 2.3.2. Caring ability inventory (CAI)

Caring ability inventory was compiled by NGOZI^[13]^ in 1990 and introduced and localized by Xu.^[14]^ It mainly starts from 3 dimensions: cognition, courage, and patience, each reflecting different psychological traits or abilities, with a total of 37 items. Using the Likert 7-point rating system, the score ranges from 1 to 7 points in the order of “completely disagree” to “completely agree,” with a total score between 37 and 259 points. Among them, a score <203.10 indicates weak caring ability; a score of 203.10 to 220.30 indicates a moderate level of caring ability; a score >220.30 indicates strong caring ability. The higher the overall score, the stronger the humanistic care ability. The Cronbach’s alpha coefficient of the scale is 0.84, the content validity is 0.78, and the reliability and validity are good.^[15]^ At present, it has been widely applied in the humanistic care ability of nursing staff.

#### 2.3.3. Emotional intelligence scale

The emotional intelligence scale adopts the revised “Emotional Intelligence Scale” by foreign scholar Schutte. This is a self-reported questionnaire consisting of 33 questions, covering 4 dimensions: emotional perception, self-emotional management, others’ emotional management, and emotional utilization. The 5-point scoring method is used to evaluate the emotional intelligence level of the respondents.^[16]^ The Chinese version of EIS was translated by Professor Wang Caikang from South China Normal University and has good reliability and validity. The Cronbach’s alpha coefficient of the Chinese scale is 0.891.^[17]^

#### 2.3.4. General self-efficacy scale

Using the general self-efficacy scale translated and revised by Wang et al,^[18]^ which covers a total of 10 questions and adopts a Likert 4-point rating system, the total score ranges from 10 to 40 points. The higher the score of the interviewee, the stronger their self-efficacy. Previous studies have confirmed that this scale performs well in evaluating adolescent self-efficacy and has high reliability and validity.^[19]^ Specifically, if the score is below 20 points, self-efficacy is at a low level; a score between 20 and 30 indicates a moderate level; a score of 30 to 40 indicates being at a high level. The Cronbach’s alpha coefficient of this scale is 0.870, indicating good reliability and validity.

### 2.4. Data collection

This study used the Questionnaire Star online survey method, with the consent of the class teacher and the distribution of a QR code for the questionnaire. The research subjects scanned the code and anonymously filled out the questionnaire. The questionnaire has a unified guiding language, including the research purpose and significance. Before filling out the questionnaire, please note the following: First, we promise to strictly protect your privacy. The questionnaire can only be filled out with the informed consent of the research subjects. In addition, all questions in the questionnaire are mandatory and require at least 300 seconds to be filled out before being successfully submitted. Each IP address can only be filled out once. After collecting the questionnaires, the exported Excel data was checked 1 by 1. 375 questionnaires were collected this time, and 8 questionnaires that did not meet the requirements were excluded (all options had the same score, obvious regularity score, inappropriate age, etc). After that, 367 valid questionnaires were obtained, with an effective recovery rate of 97.8%.

### 2.5. Data analysis

Two researchers used Excel and IBM SPSS software (version 27.0) to record and analyze raw data. Descriptive statistics, including frequency and percentage distribution, are used to describe the demographic characteristics of a sample. Use a *t*-test with 2 independent samples to infer the difference in humanistic care ability scores between 2 groups of demographic characteristics. Use 1-way analysis of variance to infer the differences in humanistic care ability scores between groups 3 and more than 3 demographic characteristics. In addition, multiple linear regression analysis was conducted to determine the influencing factors. Use variance inflation factor and tolerance test to test for multicollinearity. Determine statistical significance when *P* < .05.

### 2.6. Ethical consideration

The research plan has been approved by the Medical Ethics Committee of Inner Mongolia Medical University. All participants signed informed consent forms. The ethical principles of respect, advantage, fairness, and non-harm guide all procedures. Researchers approached participants with the help of their homeroom teacher. Participants have the right to refuse or withdraw from the study. This study uses anonymous surveys, and the survey data is only used for academic research and not for commercial purposes.

## 3. Results

### 3.1. The demographic characteristics and scores of humanistic care ability

Independent-sample *t* test and one-way analysis of variance revealed significant differences in humanistic care ability scores among nursing undergraduate students (*P* <.05) for the following factors: gender, only-child status, class committee membership, willingness to engage in nursing work, and awareness of “humanistic care” (Table 1).

**Table 1:** Demographic characteristics and the distribution of humanistic care ability (N = 367).

Variables	Frequency (%)	*X* ± *S*	(*t*/*F*)	*P*-value
Gender			2.428	.017
Male	62 (16.9)	204.98 ± 36.17		
Female	305 (83.1)	192.95 ± 32.38		
Nation			0.040	.968
Han	323 (88.0)	195.01 ± 33.38		
Others	44 (12.0)	194.80 ± 33.15		
Grade			0.393	.758
Freshman	42 (11.4)	192.45 ± 36.83		
Sophomore	79 (21.5)	198.42 ± 33.76		
Junior	131 (35.7)	194.47 ± 31.94		
Senior	115 (31.3)	194.15 ± 33.46		
Home address			–0.428	.669
Urban	176 (48.0)	176.00 ± 194.21		
Rural areas	191 (52.0)	191.00 ± 195.70		
Political outlook			0.156	.856
Party member	14 (3.8)	198.93 ± 36.27		
Member of the group	236 (64.3)	195.24 ± 31.78		
The masses	117 (31.9)	194.00 ± 36.11		
Only child			2.034	.044
Yes	97 (26.4)	201.40 ± 37.84		
No	270 (73.6)	192.68 ± 31.28		
Class cadre			3.516	<.001
Serve as	55 (15.0)	209.33 ± 33.80		
Not serving	312 (85.0)	192.46 ± 32.63		
Participate in club activities			0.172	.864
Yes	255 (69.5)	195.18 ± 33.22		
No	112 (30.5)	194.54 ± 33.65		
Personality type			2.566	.078
Export-oriented	59 (16.1)	203.86 ± 39.78		
Towards medium-sized	207 (56.4)	192.88 ± 32.23		
Introverted type	101 (27.5)	194.12 ± 30.77		
Hospitalization experience			–0.377	.706
Yes	83 (22.6)	193.77 ± 33.29		
No	284 (77.4)	195.34 ± 33.36		
Caring for patients’ experiences			0.362	.718
Yes	225 (61.3)	195.51 ± 30.38		
No	142 (38.7)	194.15 ± 37.58		
Attitude towards the nursing profession			2.613	.051
Like	119 (32.4)	201.49 ± 35.34		
Generally	222 (60.5)	191.14 ± 30.89		
Dislike	22 (6.0)	197.55 ± 41.96		
Uncertain	4 (1.1)	200.75 ± 27.15		
Willingness to engage in nursing			3.339	.011
Very willing	90 (24.5)	204.77 ± 36.95		
Basically willing	175 (47.7)	191.26 ± 28.85		
Uncertain	75 (20.4)	192.37 ± 33.81		
Basically unwilling	17 (4.6)	185.88 ± 33.04		
Very unwilling	10 (2.7)	207.20 ± 50.79		
Heard of “humanistic care” and other related topics			6.730	<.01
I’ve heard it very clearly	169 (46.0)	202.76 ± 34.86		
Having heard of it, I have a basic understanding	174 (47.4)	189.02 ± 30.39		
I have heard of it, but I am not sure	22 (6.0)	186.14 ± 28.64		
I haven’t heard of it	2 (0.5)	154.50 ± 58.69		
Self-efficacy			40.836	<.01
High level	220	206.58 ± 34.36		
Medium level	141	22.549 ± 1.89		
Low level	6	167.67 ± 19.91		

SD = standard deviation.

Specifically, male students scored slightly higher than females (204.98 ± 36.17 vs 192.95 ± 32.38); only children scored slightly higher than non-only children (201.40 ± 37.84 vs 192.68 ± 31.28); class committee members scored higher than non-members (209.33 ± 33.80 vs 192.46 ± 32.63); students willing to work in nursing scored higher (204.77 ± 36.95); and those who were very clear about “humanistic care” scored higher (202.76 ± 34.86).

### 3.2. Analysis of the current situation of humanistic care ability for nursing students

The score for humanistic care ability is (194.99 ± 33.31) points, with the highest score for patience among all dimensions at (5.86 ± 0.91) and the lowest score for courage at (4.52 ± 1.36). The general information and humanistic care ability scores of the survey subjects are shown in Table 2.

**Table 2:** Scores of humanistic care ability, emotional intelligence, and self-efficacy dimensions for nursing students.

Variables	Number of items	Lowest score	Highest score	Score, mean (SD)	Item average score, mean (SD)
Humanistic care ability	37	88	259	194.99 ± 33.31	5.27 ± 0.90
Cognition	14	31	98	77.56 ± 13.87	5.54 ± 0.99
Courage	13	19	91	58.79 ± 17.71	4.52 ± 1.36
Patience	10	23	70	58.63 ± 9.07	5.86 ± 0.91
Emotional intelligence level	33	66	165	131.38 ± 20.09	3.98 ± 0.61
Emotional perception	12	24	60	46.53 ± 7.57	3.88 ± 0.63
Self-emotional management	8	16	40	31.71 ± 4.88	3.96 ± 0.61
Emotional management of others	6	12	30	24.30 ± 3.95	4.05 ± 0.66
Emotional utilization	7	14	35	28.84 ± 4.48	4.12 ± 0.64
Self-efficacy level	10	15	40	30.27 ± 6.30	–

SD = standard deviation.

### 3.3. Multiple linear regression analysis and multicollinearity test

Perform multiple linear regression analysis with the total score of humanistic care ability for nursing students as the dependent variable. Variables with statistical significance in univariate analysis results are used as independent variables, and their values are shown in Table 3. The collinearity diagnosis of the final regression model indicated that the tolerance for all independent variables was >0.2 (with an actual range of 0.423–0.992), and the variance inflation factor was <5 (with an actual range of 1.008–2.363). The results showed that gender, emotional intelligence, and self-efficacy entered the regression equation, explaining 56.1% of the total variance. Table 4 provides more detailed information.

**Table 3:** Independent variable assignment.

Independent variable	Assignment
Gender	Men = 1, Women = 2
Only child	No = 0, Yes = 1
Class cadre	Not serving = 0, serve as = 1
Willingness to engage in nursing	Very unwilling = 0, Basically unwilling = 1, Uncertain = 2, Basically willing = 3, Very willing = 4
Heard of “humanistic care” and other related topics	I haven’t heard of it = 0, I have heard of it, but I am not sure = 1, Having heard of it, I have a basic understanding = 2, I’ve heard it very clearly = 3
Self-efficacy	Low level = 1, Medium level = 2, High level = 3

**Table 4:** Multivariate linear regression analysis of factors influencing the humanistic care ability of nursing students(N = 367).

Independent variable	*B*	SE	β	*T*	*P*	Collinearity statistics		
						Tolerance		VIF
(Constant)	46.786	9.900	–	4.726	<.001	–	–	
Emotional intelligence	1.089	0.088	0.657	12.335	<.001	0.423	2.363	
Gender	−6.591	3.087	−0.074	-2.135	.033	0.992	1.008	
Self-efficacy	0.568	0.281	0.107	2.022	.044	0.425	2.352	

Dependent variable: Ability to provide humanistic care.

*F* = 156.835, *P* < .001, *R*^2^ = 0.564, adjusted *R*^2^ = 0.561.

SE = standard error, VIF = variance inflation factor.

### 3.4. Correlation analysis between humanistic care ability and emotional intelligence of intern nursing students

Pearson correlation analysis showed that the total score of humanistic care ability and emotional intelligence of intern nursing students were positively correlated (*r* = 0.745, *P* <.01), as shown in Table 5.

**Table 5:** Correlation analysis between humanistic care ability and emotional intelligence of intern nursing students (*r*-value).

Correlation	1	2	3	4	5	6	7	8	9
Average score of humanistic care	1								
Average score of cognition	.863**	1							
Average score of courage	.789**	.397**	1						
Average score of patience	.812**	.866**	.337**	1					
Average score of emotional perception	.755**	.774**	.464**	.685**	1				
Average score of self-emotional management	.758**	.769**	.446**	.736**	.919**	1			
Average score of others’ emotional management	.668**	.783**	.269**	.731**	.881**	.892**	1		
Average score of emotional application	.648**	.762**	.231**	.764**	.873**	.906**	.932**	1	
Average score of emotional intelligence	.745**	.802**	.388**	.751**	.968**	.967**	.953**	.955**	1

1. Average score of humanistic care.

2. Average score of cognition.

3. Average score of courage.

4. Average score of patience.

5. Average score of emotional perception.

6. Average score of self-emotional management.

7. Average score of others’ emotional management.

8. Average score of emotional application.

9. Average score of emotional intelligence.

** At the 0.01 level (double-tailed), the correlation is significant.

## 4. Discussion

### 4.1. Current situation of humanistic care ability among undergraduate nursing students

The results of this study showed that the total score of nursing students’ humanistic care ability was (194.99 ± 33.31) points, which was higher than the research score of Wang et al^[20]^ (184.63 ± 19.88) points. Compared with the total score of humanistic care ability and each dimension norm of nursing students in Chinese Mainland in the study of Chen^[21]^ in China, the scores of humanistic care ability and cognitive dimension of undergraduate nursing students were higher than the domestic norm, with a statistically significant difference (*P* <.01); the differences in other dimensions were not statistically significant (*P* >.05). See Table 6. The humanistic care ability of undergraduate nursing students is higher than the domestic norm, which may be due to the strengthening of medical humanities education reform and curriculum ideological and political construction in medical colleges in recent years, which has had a positive impact on enhancing the humanistic care ability of medical students. It may also be related to the fact that undergraduate nursing students have not yet entered clinical internships and have not personally experienced the interaction process with patients and their families. However, it is still lower than the foreign norm, and the humanistic care ability of undergraduate nursing students needs further improvement.

**Table 6:** Comparison of scores of humanistic care ability among undergraduate nursing students with the domestic norm.

	Undergraduate group (n = 367)	Domestic norm (n = 8574)	*T*	*P*
Total score of humanistic care	194.99 ± 33.31	191.04 ± 19.49	2.270	.024
Cognitive	77.56 ± 13.87	73.82 ± 9.22	5.173	<.001
Courage	58.79 ± 17.71	59.31 ± 10.43	-0.565	.572
Patience	58.63 ± 9.07	58.51 ± 6.17	0.264	.792

In this study, patience was the highest scoring dimension with a score of (5.86 ± 0.91), while courage was the lowest scoring dimension with a score of (3.05 ± 0.66), which is consistent with the research results of Jie^[22]^ and Chen.^[23]^ The reason for this may be that the proportion of female nursing students is much higher than that of male students, and female students are more patient. The courage to care usually comes from practical experience, and the improvement of courage in the process of caring usually requires corresponding practice to support it.^[24]^ The current humanistic care education in our country is mostly theoretical teaching, with monotonous course forms and a lack of humanistic practice. This often leads to the separation of nursing basic professional courses from humanistic care education, forming a “parallel teaching,” which is not conducive to cultivating students’ courage to care.^[25]^ Therefore, colleges and universities need to actively explore effective teaching methods, and in curriculum design, students should be exposed to clinical practice as early as possible. The clinical practice of “early clinical and multi-clinical” can enable students to perceive humanistic care more intuitively and have a deeper understanding.

### 4.2. Influence of demographic factors on humanistic care ability

#### 4.2.1. Gender difference

Male students scored higher than females (204.98 ± 36.17 vs 192.95 ± 32.38), contrary to most previous studies. This may be due to sample bias (males only 16.7%, possible self-selection), the “minority group” effect in nursing, and gender differences in clinical internships (e.g., male students gaining confidence in emergency/Intensive care unit settings).

#### 4.2.2. Only-child status

Only children scored higher than non-only children (201.40 ± 37.84 vs 192.68 ± 31.28), also contrary to most research. Possible explanations include greater family resource concentration, proactive peer socialization, and democratic parenting styles in only-child families.

#### 4.2.3. Class committee membership

Class committee members scored higher than non-members (209.33 ± 33.80 vs 192.46 ± 32.63), consistent with existing studies. This may be due to more practical exercise opportunities, a stronger sense of responsibility, and leadership development.

### 4.3. Analysis of factors influencing the humanistic care ability of undergraduate nursing students

In addition to the demographic factors discussed above, willingness to engage in nursing work and receiving humanistic care training were also found to significantly influence humanistic care ability. Furthermore, there is a positive correlation between the humanistic care ability and the emotional intelligence level of undergraduate nursing students. The cognitive and patient dimensions are highly correlated with emotional intelligence level, with correlation coefficients of 0.802 and 0.751, respectively (*P* <.001). The higher the emotional intelligence level, the stronger the humanistic care ability, which is consistent with the research results of Lina et al.^[26]^ The results of multiple linear regression analysis indicate that the lower the level of emotional intelligence, the lower the humanistic care ability of nursing students; The lower the self-efficacy, the lower the humanistic care ability of nursing students. This is similar to previous research results.^[27]^

#### 4.3.1. Emotional intelligence

The research results indicate that the emotional intelligence level of undergraduate nursing students is positively correlated with their overall humanistic care ability; that is, the higher their emotional intelligence, the stronger their humanistic care ability. This is consistent with the research results of Lina^[26]^ and Zhou.^[28]^ The reason may be that nursing students with high levels of emotional intelligence are usually better at managing their own emotions and are able to remain calm and rational in the face of pressure and challenges.^[29]^ This self-emotional management ability enables them to better cope with various complex situations in nursing work and avoid affecting the quality of care for patients due to individual emotional fluctuations. Nursing students with high emotional intelligence are able to keenly perceive the emotional changes of others and empathize with their feelings. In addition, nursing students with high emotional intelligence usually have strong communication and interpersonal skills.^[30]^ They can effectively communicate with patients and their families, establish harmonious nurse–patient relationships, promote the smooth progress of nursing work, and improve patient satisfaction. Therefore, nursing educators should strengthen the exploration and cultivation of emotional intelligence in nursing students. This can be achieved by integrating the cultivation of emotional intelligence into professional talent training programs, integrating it into medical fundamentals, nursing courses, and clinical nursing practice teaching. At the same time, nursing students can also be helped to master corresponding social communication skills and enhance their emotional intelligence and humanistic care abilities through vocational core competency training, club activity expansion, and other means.

#### 4.3.2. Self-efficacy

The results indicate that there is a close relationship between the humanistic care ability of nursing students and their self-efficacy, which is consistent with the research of Liu et al.^[31]^ The reason may be that nurses with higher levels of self-efficacy also have higher levels of occupational benefits. The self-efficacy of nursing students directly affects their attitudes, behaviors, and ultimately nursing outcomes when facing nursing tasks.^[32]^ A student with strong self-efficacy is confident in their abilities and is more likely to approach nursing challenges with a positive and proactive attitude,^[33]^ thereby continuously improving their humanistic care skills in practice. Therefore, undergraduate teachers should help students clarify their learning goals and the abilities they will possess for future careers in this industry, and develop personal development plans to enhance their confidence; encourage students to review their successful experiences, whether it be academic achievements or personal challenges, to make them aware of their ability to overcome difficulties, showcase industry leaders, outstanding alumni or classmates, and let them understand that significant results can be achieved through unremitting efforts, thereby enhancing their self-efficacy.

### 4.4. Strengths and limitations

This study provides relevant evidence for improving the humanistic care ability of nursing undergraduate students. These findings provide necessary data for developing effective intervention plans. Although this study has been carefully designed, certain aspects still need improvement. First, the limitation of the study is that it only focuses on undergraduate nursing students and does not include comparisons with nursing students with other educational backgrounds. Future research should include nursing students with different educational levels for comparative analysis to more comprehensively evaluate the role of nursing education in shaping humanistic care abilities. Second, due to time and manpower constraints, the sample size of this study is relatively small, and this conclusion may not be representative enough. Future research should expand the sample size and improve sample representativeness. Finally, this study evaluated the humanistic care ability level of nursing students based on self-reported questionnaires, and the results may have biases. Therefore, in the future, we can combine qualitative interview methods to explore and analyze the factors that affect the humanistic care ability of nursing students.

## 5. Conclusion

In summary, the level of humanistic care ability for nursing students is at a moderate level, but further improvement is needed. The factors that affect humanistic care ability include gender, emotional intelligence level, and self-efficacy. The characteristics of these factors provide valuable insights for nursing education personnel. Nursing educators should regard the cultivation of emotional intelligence and self-efficacy in nursing students as an important task, to promote the continuous improvement of their humanistic care abilities, lay a solid foundation for building harmonious nurse–patient relationships and providing high-quality nursing services, thereby improving clinical nursing services and avoiding violent injuries to doctors.

## Acknowledgments

The researchers thank all the participants for their valuable time in this study. We are also grateful to the schools and teachers for their support and assistance during the study.

## Author contributions

**Conceptualization:** Jia Li, Hai-xia Xu, Min Li, Yong-Li Wang.

**Data curation:** Jia Li, Hai-xia Xu, Min Li, Yong-Li Wang, Ling-ling Li, Te-er Ba.

**Formal analysis:** Jia Li, Hai-xia Xu, Min Li, Yong-Li Wang.

**Investigation:** Jia Li, Hai-xia Xu, Min Li, Yong-Li Wang, Ling-ling Li, Te-er Ba.

**Methodology:** Jia Li, Hai-xia Xu, Min Li, Yong-Li Wang.

**Resources:** Jia Li.

**Project administration:** Hai-xia Xu.

**Software:** Jia Li, Hai-xia Xu, Min Li, Yong-Li Wang.

**Validation:** Hai-xia Xu.

**Writing – original draft:** Jia Li.

**Writing – review & editing:** Jia Li, Hai-xia Xu, Min Li, Yong-Li Wang.

## References

[1] Radwin LE Cabral HJ Seibert MN. Patient-centered care in primary care scale: pilot development and psychometric assessment. J Nurs Care Qual. 2019;34:34–9. 30045359 10.1097/NCQ.0000000000000341

[2] Cai R Tang J Deng C. Violence against health care workers in China, 2013-2016: evidence from the national judgment documents. Hum Resour Health. 2019;17:103. 31878939 10.1186/s12960-019-0440-y PMC6933725

[3] Lu L Dong M Wang SB. Prevalence of workplace violence against health-care professionals in China: a comprehensive meta-analysis of observational surveys. Trauma Violence Abuse. 2020;21:498–509. 29806556 10.1177/1524838018774429

[4] Ministry of Education of the People’s Republic of China. Guidelines for the construction of ideological and political education in higher education curriculum. 2020. http://www.moe.gov.cn/srcsite/A08/s7056/202006/t20200603_462437.html

[5] Li L Wang F. Application of experiential teaching in the training of humanistic care ability for cardiovascular nurses. Tianjin Nurs. 2022;30:354–6.

[6] Sharon D Grinberg K. Does the level of emotional intelligence affect the degree of success in nursing studies? Nurse Educ Today. 2018;64:21–6. 29454875 10.1016/j.nedt.2018.01.030

[7] National Health Commission of the People’s Republic of China. China has 5.63 mln registered nurses. Xinhua News. 2024. https://english.news.cn/20240511/7094a1a87a554247b234a6dad5222cf1/c.html

[8] Moudatsou M Stavropoulou A Philalithis A Koukouli S. The role of empathy in health and social care professionals. Healthcare (Basel). 2020; 8:26. 32019104 10.3390/healthcare8010026 PMC7151200

[9] Zhang FJ Zhang HX Liu YL. Analysis of the current situation and influencing factors of patients’ perception of nursing humanistic care in 30 provincial hospitals. Chin J Nurs. 2024;59:324–30.

[10] Hua QQ Dai XY Yin XH. Exploring the path of cultivating humanistic care ability in medical students from the perspective of narrative medicine. J Med Philos. 2022;43:68–72.

[11] Xuel L Peng ZY Wang R. The correlation between medical narrative ability, humanistic care ability, and professional identity of intern nursing students. Evid Based Nurs. 2024;10:1126–8.

[12] Ni P Chen JL Liu N. The sample size estimation in quantitative nursing research. Chin J Nurs. 2010;45:378–80.

[13] Nkongho N. Measurement of nursing outcomes [M]. Springer,1990:3–16.

[14] Xu J. A study on the care ability of hospital nursing staff. Huazhong Univ Sci Technol. 2008.

[15] Xu J Liu YL. Investigation and analysis of the caring ability of clinical nursing staff. J Nurs. 2008;23:16–8.

[16] Schutte NS Malouff JM Hall LE. Development and validation of a measure of emotional intelligence. Personality Individual Differences. 1998;25:167–77.

[17] Caikang W. A study on the emotional intelligence and related personality factors of juvenile delinquences. Chin J Mental Health. 2002;16:566–567+565.

[18] Wang CK Hu ZF Liu Y. A study on the reliability and validity of general self efficacy scale. Appl Psychol. 2001;7:37–40.

[19] Zheng XL Zhao W. The relationship between online altruistic behavior and hope in middle school students: the mediating role of self efficacy and self esteem. Psychol Develop Educ. 2015;31:428–36.

[20] Wang Y Zhang X Xie Q Zhou H Cheng L. Humanistic caring ability of midwifery students in China and its associated factors: a multi-centre cross-sectional study. Nurse Educ Today. 2022;111:105276. 35131563 10.1016/j.nedt.2022.105276

[21] Chen Y. A survey and intervention study on the humanistic care ability of nursing college students. Southern Med Univ. 2017.

[22] Jie H. Research on the current situation and influencing factors of humanistic care ability of vocational nursing students. Shandong Univ. 2021.

[23] Chen SR. The current status and correlative factors of humanistic care ability in the standardized training nurses. Henan University; 2019.

[24] Liu Y Liu AZ. Analysis of the current situation and related factors of humanistic care ability of college nursing students. Contemp Nurse. 2016:158–161.

[25] Li N Liu C Wei JL. Thoughts on the integration and optimization of the nursing humanities courses’contents. Int J Nurs. 2016;35:583585–608.

[26] Lina M Qin G Yang L. Mediating effects of emotional intelligence on the relationship between empathy and humanistic care ability in nursing students: a cross-sectional descriptive study. Medicine (Baltim). 2022;101:e31673. 10.1097/MD.0000000000031673 PMC9678621 36401377

[27] Bo X Bin C. A study on the correlation between nurses’ occupational benefits and self efficacy. Contemp Nurses (Firs Edition). 2021;28:16–9.

[28] Zhou WQ Deng JY Dong ZY. Factors influencing the humanistic care ability of clinical nurses. Hebei Pharm. 2024;46:1730–3.

[29] Li Q. Research on individual emotions, improvisation, and employee innovation performance. Anhui Univ. 2020.

[30] Zhang XL Geng MF Di Q. Potential categories of emotional intelligence in clinical nurses and their relationship with communication skills. Nurs Res. 2024;38:3227–34.

[31] Liu X Li C Yan X Shi B. Psychological capital has a positive correlation with humanistic care ability among nurses. Front Psychol. 2022;13:955627. 36186317 10.3389/fpsyg.2022.955627 PMC9524352

[32] Zhong XL Liu XZ Sheng Y. The effect of the humanistic care teaching model on nurse patient conflict and nurse turnover intention in a pediatric outpatient department: results of a randomized trial. Transl Pediatr. 2021;10:2016–23. 34584871 10.21037/tp-21-214 PMC8429853

[33] Zhang TY. Research on the current status and correlation of occupational benefits and self efficacy among intern nursing students. Shandong Univ. 2018.

